# Associations between the number of antipsychotics prescribed and metabolic parameters in Japanese patients with schizophrenia

**DOI:** 10.1002/pcn5.28

**Published:** 2022-07-07

**Authors:** Yuichiro Watanabe, Shin Ono, Takuro Sugai, Yutaro Suzuki, Manabu Yamazaki, Norio Sugawara, Norio Yasui‐Furukori, Kazutaka Shimoda, Takao Mori, Yuji Ozeki, Hiroshi Matsuda, Kurefu Okamoto, Toyoaki Sagae, Toshiyuki Someya

**Affiliations:** ^1^ Department of Psychiatry, Graduate School of Medical and Dental Sciences Niigata University Niigata Japan; ^2^ Murakamihamanasu Hospital Murakami Niigata Japan; ^3^ Japan Psychiatric Hospital Association Tokyo Japan; ^4^ Department of Psychiatry, School of Medicine Dokkyo Medical University Mibu Tochigi Japan; ^5^ Department of Psychiatry Shiga University of Medical Science Otsu Shiga Japan; ^6^ Department of Health and Nutrition, Faculty of Health and Nutrition Yamagata Prefectural Yonezawa University of Nutrition Sciences Yonezawa Yamagata Japan

**Keywords:** antipsychotic polypharmacy, aripiprazole, blood pressure, body mass index, side‐effects

## Abstract

**Aim:**

There is little evidence on the effects of antipsychotic polypharmacy on metabolic parameters in patients with schizophrenia. Thus, this cross‐sectional study explored the associations between the number of antipsychotics prescribed and metabolic parameters in Japanese patients with schizophrenia.

**Methods:**

We obtained metabolic parameter data from 19,675 patients with schizophrenia. Of these, 1380 (7.0%), 8422 (42.8%), 6326 (32.2%), and 3547 (18.0%) were treated with none, one, two, and three or more antipsychotics, respectively. We compared eight metabolic parameters among the four groups using univariate analyses. We then performed multiple regression analysis to assess the effect of the number of antipsychotics prescribed on metabolic parameters after controlling for the effects of age, sex, type of care (outpatient/inpatient), chlorpromazine‐equivalent dose, and antipsychotic type (aripiprazole, olanzapine, and risperidone).

**Results:**

There were significant differences in body mass index (BMI), systolic and diastolic blood pressure (dBP), total cholesterol, low‐density lipoprotein cholesterol, and triglycerides among the four groups. The multiple regression analysis showed that the number of antipsychotics prescribed was significantly correlated with BMI and dBP (standardized regression coefficient = 0.031 and 0.026, respectively).

**Conclusion:**

Our results suggested that the number of antipsychotics prescribed adversely affects BMI and dBP. Clinicians should avoid inappropriate antipsychotic polypharmacy, especially polypharmacy involving three or more antipsychotics.

## Graphical abstract

Our results suggested that the number of antipsychotics prescribed adversely affects body mass index and diastolic blood pressure. Clinicians should avoid inappropriate antipsychotic polypharmacy, especially polypharmacy involving three or more antipsychotics.

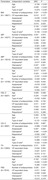

## INTRODUCTION

Although antipsychotic polypharmacy is frequently used in clinical practice, the treatment guidelines for schizophrenia recommend antipsychotic monotherapy.^1^, ^2^ In Japan, approximately half of all patients with schizophrenia receive antipsychotic polypharmacy.^3^, ^4^ The benefits and risks of antipsychotic polypharmacy are controversial.^1^, ^2^ For example, a recent meta‐analysis reported that mortality risk does not differ between antipsychotic polypharmacy and monotherapy,^5^ whereas antipsychotic polypharmacy may be associated with adverse drug events.^6^

Metabolic syndromes and metabolic abnormalities are major side‐effects of antipsychotic use.^7^ However, there is little evidence on the effects of antipsychotic polypharmacy on metabolic syndromes and metabolic parameters in patients with schizophrenia.^8^ Although several cross‐sectional studies have examined the associations among antipsychotic polypharmacy and metabolic syndromes and metabolic parameters in patients with schizophrenia, findings are inconsistent, which may be attributed to the small sample sizes (*n* = 118–543).^9^, ^10^, ^11^, ^12^, ^13^ Using univariate analysis, Correll et al.^10^ found that patients treated with antipsychotic polypharmacy had higher rates of metabolic syndromes than those undergoing antipsychotic monotherapy. However, this result became non‐significant after controlling for relevant variables using multiple regression. Therefore, examining the effects of antipsychotic polypharmacy and relevant variables (e.g., age and sex) on metabolic parameters using both univariate and multivariate analyses is valuable.

This cross‐sectional study aimed to assess the associations between the number of antipsychotics prescribed and metabolic parameters in Japanese patients with schizophrenia.

## METHODS

### Participants

This study was approved by the Institutional Review Board of the Japan Psychiatric Hospital Association. All participants provided written informed consent.

This study was part of the Joint Project on Antipsychotic Treatment and Physical Risk established by the Japan Psychiatric Hospital Association and the Japanese Society of Clinical Neuropsychopharmacology.^14^, ^15^, ^16^, ^17^ We conducted a questionnaire survey across 520 outpatient and 247 inpatient facilities belonging to the Japan Psychiatric Hospitals Association between January 2012 and July 2014. Patients with schizophrenia were recruited as described previously.^14^, ^15^, ^16^, ^17^ Briefly, patients were diagnosed according to the *Diagnostic and Statistical Manual of Mental Disorders*, fourth edition, text revision^18^ criteria, or the *International Statistical Classification of Diseases and Related Health Problems*, Version 10^19^ criteria. We excluded individuals who were under 20 years old.

### Measurements

We compiled a brief questionnaire that encompassed age, sex, type of care (outpatient/inpatient), prescribed dose of antipsychotics, height and weight, systolic (sBP) and diastolic blood pressure (dBP), total cholesterol (TC), low‐density lipoprotein cholesterol (LDL‐C), high‐density lipoprotein cholesterol (HDL‐C), triglycerides (TG), and fasting blood sugar (FBS) level. Body mass index (BMI) was calculated as the ratio of body weight to height (kg/m^2^). We calculated chlorpromazine (CP)‐equivalent doses using established conversion formulas.^20^

### Statistical analysis

Participants were assigned to four groups according to the number of antipsychotics prescribed: none, one, two, and three or more. We compared age, sex, type of care, CP‐equivalent dose, type of antipsychotics prescribed (aripiprazole, olanzapine, and risperidone), and eight metabolic parameters (BMI, sBP, dBP, TC, HDL‐C, LDL‐C, TG, and FBS) among the four groups using analyses of variance (ANOVAs) and χ^2^ tests. The antipsychotic type has been shown to have an effect on metabolic parameters.^10^, ^21^, ^22^ Olanzapine, risperidone, and aripiprazole have the poorest, moderate, and safest metabolic side‐effect profiles, respectively.^21^ These were the three most commonly prescribed antipsychotics in our study. Therefore, we included aripiprazole, olanzapine, and risperidone in the analyses. For the univariate analyses, the significance level was set at *p* < 0.003 based on Bonferroni correction for 15 tests. When significant differences were found among the four groups in the ANOVA, we performed post hoc tests to identify significant differences between groups with Bonferroni correction.

We then performed multiple regression analysis to assess the effects of potential predictors (age, sex, type of care, number of antipsychotics prescribed, CP‐equivalent dose, and antipsychotic type) on the eight metabolic parameters. Men and women were coded as 1 and 2, respectively. Outpatients and inpatients were coded as 1 and 2, respectively. The prescription of 0, 1, 2, and 3 or more antipsychotics was coded as 0, 1, 2, and 3, respectively. Not taking and taking aripiprazole, olanzapine, and risperidone were coded as 0 and 1, respectively. For the multiple regression analysis, the significance level was set at *p* < 0.006 based on Bonferroni correction for eight tests.

We used SPSS for Windows Version 19.0 (IBM Japan) for all statistical analyses.

## RESULTS

The study participants comprised 19,675 patients with schizophrenia. Of these, 1380 (7.0%), 8422 (42.8%), 6326 (32.2%), and 3547 (18.0%) were treated with none (Group 0), one (Group 1), two (Group 2), and three or more (Group 3) antipsychotics, respectively (Table 1). There were significant differences in age, sex, type of care, CP‐equivalent dose, antipsychotic type (aripiprazole, olanzapine, and risperidone), and six of the eight metabolic parameters (i.e., BMI, sBP, dBP, TC, LDL‐C, and TG) among the four groups. Age was highest in Group 0, followed by Groups 1, 2, and 3. In contrast, CP‐equivalent dose was highest in Group 3, followed by Groups 2, 1, and 0. BMI was significantly higher in Groups 1, 2, and 3 than in Group 0. BMI was significantly higher in Group 2 than in Group 1. The sBP was significantly higher in Groups 0 and 1 than in Group 3. The dBP was significantly higher in Group 3 than in Group 1. TC was significantly higher in Groups 1 and 2 than in Group 3. LDL‐C and TG were significantly higher in Group 1 than in Group 3.

**Table 1:** Number of antipsychotics prescribed and metabolic parameters

Parameters	The number of antipsychotics				*p*						
	0	1	2	3 or more	Overall	Post‐hoc					
						0 versus 1	0 versus 2	0 versus 3 or more	1 versus 2	1 versus 3 or more	2 versus 3 or more
*N*	1380 (7.0%)	8422 (42.8%)	6326 (32.2%)	3547 (18.0%)	–	–	–	–	–	–	–
Age (years)	61.6 ± 14.3	59.3 ± 14.0	57.0 ± 13.0	54.6 ± 12.4	<0.001^a^	<0.001	<0.001	<0.001	<0.001	<0.001	<0.001
Sex	–	–	–	–	<0.001^b^	–	–	–	–	–	–
Men	716 (51.9%)	4355 (51.7%)	3556 (56.2%)	2045 (57.7%)	–	–	–	–	–	–	–
Women	664 (48.1%)	4067 (48.3%)	2770 (43.8%)	1502 (42.3%)	–	–	–	–	–	–	–
Type of care	–	–	–	–	<0.001^b^	–	–	–	–	–	–
Outpatient	336 (24.3%)	2687 (31.9%)	1653 (26.1%)	765 (21.6%)	–	–	–	–	–	–	–
Inpatient	1044 (75.7%)	5735 (68.1%)	4673 (73.9%)	2782 (78.4%)	–	–	–	–	–	–	–
CP‐equivalent dose (mg/day)	0 (*N* = 1380)	401.9 ± 296.4 (*N* = 8421)	754.5 ± 477.7 (*N* = 6325)	1290.0 ± 754.9 (*N* = 3545)	<0.001^a^	<0.001	<0.001	<0.001	<0.001	<0.001	<0.001
Type of antipsychotics	–	–	–	–	–	–	–	–	–	–	–
Aripiprazole	–	913 (10.8%)	726 (11.5%)	482 (13.6%)	<0.001^b^	–	–	–	–	–	–
The others	–	7509 (89.2%)	5600 (88.5%)	3065 (86.4%)	–	–	–	–	–	–	–
Olanzapine	–	1696 (20.1%)	1630 (25.8%)	1249 (35.2%)	<0.001^b^	–	–	–	–	–	–
The others	–	6726 (79.9%)	4696 (74.2%)	2298 (64.8%)	–	–	–	–	–	–	–
Risperidone	–	2440 (29.0%)	2626 (41.5%)	1776 (50.1%)	<0.001^b^	–	–	–	–	–	–
The others	–	5982 (71.0%)	3700 (58.5%)	1771 (49.9%)	–	–	–	–	–	–	–
BMI (kg/m^2^)	22.5 ± 4.5 (*N* = 1380)	23.0 ± 4.5 (*N* = 8422)	23.3 ± 4.3 (*N* = 6326)	23.2 ± 4.3 (*N* = 3547)	<0.001^a^	<0.001	<0.001	<0.001	<0.001	0.400	0.838
sBP (mmHg)	123.1 ± 17.5 (*N* = 1235)	122.4 ± 17.9 (*N* = 7773)	122.2 ± 17.8 (*N* = 5981)	121.0 ± 17.2 (*N* = 3392)	<0.001^a^	1.000	0.701	0.003	1.000	<0.001	0.012
dBP (mmHg)	75.2 ± 12.0 (*N* = 1223)	75.0 ± 12.3 (*N* = 7748)	75.6 ± 12.2 (*N* = 5959)	76.3 ± 12.4 (*N* = 3384)	<0.001^a^	1.000	1.000	0.051	0.024	<0.001	0.045
TC (mg/dL)	183.1 ± 41.7 (*N* = 759)	184.8 ± 39.1 (*N* = 5213)	183.0 ± 37.4 (*N* = 3923)	179.0 ± 36.3 (*N* = 2302)	<0.001^a^	1.000	1.000	0.059	0.164	<0.001	<0.001
HDL‐C (mg/dL)	56.1 ± 23.0 (*N* = 561)	55.1 ± 20.2 (*N* = 3472)	55.0 ± 18.6 (*N* = 2630)	55.7 ± 18.3 (*N* = 1546)	0.474^a^	–	–	–	–	–	–
LDL‐C (mg/dL)	109.2 ± 35.4 (*N* = 612)	110.6 ± 35.3 (*N* = 3526)	108.8 ± 50.0 (*N* = 2779)	104.6 ± 32.6 (*N* = 1670)	<0.001^a^	1.000	1.000	0.096	0.511	<0.001	0.005
TG (mg/dL)	111.6 ± 68.5 (*N* = 910)	114.6 ± 76.8 (*N* = 5961)	112.4 ± 74.5 (*N* = 4487)	107.7 ± 71.9 (*N* = 2548)	0.002^a^	1.000	1.000	1.000	0.877	<0.001	0.062
FBS (mg/dL)	102.8 ± 40.2 (*N* = 480)	99.6 ± 34.6 (*N* = 3426)	98.7 ± 29.3 (*N* = 2550)	98.3 ± 32.5 (*N* = 1360)	0.054^a^	–	–	–	–	–	–

*Note*: Data are expressed as means ± standard deviations. The level of significance was set at an overall *p* < 0.003 based on Bonferroni correction for 15 tests. We applied Bonferroni correction to post‐hoc tests to identify significant differences between groups.

Abbreviations: BMI, body mass index; CP, chlorpromazine; dBP, diastolic blood pressure; FBS, fasting blood sugar; HDL‐C, high‐density lipoprotein cholesterol; LDL‐C, low‐density lipoprotein cholesterol; sBP, systolic blood pressure; TC, total cholesterol; TG, triglycerides.

^a^ Calculated using an analysis of variance.

^b^ Calculated using a χ^2^ test.

The multiple regression analyses showed that age (standardized regression coefficient [SRC] = −0.164), sex (SRC = 0.028), type of care (SRC = −0.275), the number of antipsychotics prescribed (SRC = 0.031), aripiprazole (SRC = −0.035), and olanzapine (SRC = −0.046) were significantly correlated with BMI (Table 2). Age (SRC = −0.055), sex (SRC = −0.086), type of care (SRC = −0.118), the number of antipsychotics prescribed (SRC = 0.026), olanzapine (SRC = 0.027), and risperidone (SRC = −0.031) were significantly correlated with dBP. The number of antipsychotics prescribed was not significantly correlated with sBP, TC, HDL‐C, LDL‐C, TG, or FBS after controlling for the effects of age, sex, type of care, CP‐equivalent dose, aripiprazole, olanzapine, and risperidone.

**Table 2:** Multiple regression analysis for the effects of the independent variables on metabolic parameters

Parameters	Independent variables	SRC	*p*
BMI (*N* = 19,671)	Age	−0.164	<0.001
	Sex^a^	0.028	<0.001
	Type of care^b^	−0.275	<0.001
	Number of antipsychotics	0.031	<0.001
	CP‐equivalent dose	0.012	0.177
	Aripiprazole^c^	−0.035	<0.001
	Olanzapine^c^	−0.046	<0.001
	Risperidone^c^	0.015	0.033
sBP (*N* = 18,377)	Age	0.135	<0.001
	Sex^a^	−0.083	<0.001
	Type of care^b^	−0.194	<0.001
	Number of antipsychotics	0.001	0.875
	CP‐equivalent dose	−0.001	0.880
	Aripiprazole^c^	0.009	0.241
	Olanzapine^c^	0.006	0.449
	Risperidone^c^	−0.021	0.007
dBP (*N* = 18,310)	Age	−0.055	<0.001
	Sex^a^	−0.086	<0.001
	Type of care^b^	−0.118	<0.001
	Number of antipsychotics	0.026	0.005
	CP‐equivalent dose	0.010	0.305
	Aripiprazole^c^	−0.017	0.025
	Olanzapine^c^	0.027	0.001
	Risperidone^c^	−0.031	<0.001
TC (*N* = 12,193)	Age	0.017	0.070
	Sex^a^	0.224	<0.001
	Type of care^b^	−0.231	<0.001
	Number of antipsychotics	−0.005	0.630
	CP‐equivalent dose	−0.023	0.042
	Aripiprazole^c^	−0.019	0.033
	Olanzapine^c^	0.040	<0.001
	Risperidone^c^	−0.022	0.017
HDL‐C (*N* = 8207)	Age	0.009	0.408
	Sex^a^	0.234	<0.001
	Type of care^b^	−0.032	0.005
	Number of antipsychotics	0.004	0.745
	CP‐equivalent dose	0.011	0.447
	Aripiprazole^c^	0.022	0.043
	Olanzapine^c^	0.006	0.619
	Risperidone^c^	0.012	0.289
LDL‐C (*N* = 8587)	Age	0.004	0.745
	Sex^a^	0.101	<0.001
	Type of care^b^	−0.121	<0.001
	Number of antipsychotics	0.002	0.867
	CP‐equivalent dose	−0.069	<0.001
	Aripiprazole^c^	0.007	0.500
	Olanzapine^c^	0.046	<0.001
	Risperidone^c^	−0.002	0.847
TG (*N* = 13,903)	Age	−0.071	<0.001
	Sex^a^	−0.066	<0.001
	Type of care^b^	−0.222	<0.001
	Number of antipsychotics	0.011	0.291
	CP‐equivalent dose	−0.034	0.002
	Aripiprazole^c^	−0.021	0.014
	Olanzapine^c^	−0.013	0.131
	Risperidone^c^	−0.046	<0.001
FBS (*N* = 7815)	Age	0.102	<0.001
	Sex^a^	−0.040	<0.001
	Type of care^b^	−0.260	<0.001
	Number of antipsychotics	0.035	0.011
	CP‐equivalent dose	−0.011	0.442
	Aripiprazole^c^	−0.025	0.026
	Olanzapine^c^	−0.110	<0.001
	Risperidone^c^	−0.012	0.319

*Note*: The level of significance was set at *p* < 0.006 based on Bonferroni correction for eight tests.

Abbreviations: BMI, body mass index; CP, chlorpromazine; dBP, diastolic blood pressure; FBS, fasting blood sugar; HDL‐C, high‐density lipoprotein cholesterol; LDL‐C, low‐density lipoprotein cholesterol; sBP, systolic blood pressure; SRC, standardized regression coefficient; TC, total cholesterol; TG, triglycerides.

^a^ Reference is men.

^b^ Reference is outpatient.

^c^ Reference is not taking the antipsychotic.

## DISCUSSION

The rate of antipsychotic polypharmacy is considered to be unnecessarily high in Japan.^2^ In this study, 9873 of 19,675 (50.2%) patients with schizophrenia had been prescribed multiple antipsychotics. Notably, 3547 (18.0%) patients were treated with three or more antipsychotics. Moreover, recent studies conducted in Japan have reported that approximately half of all patients with schizophrenia are undergoing antipsychotic polypharmacy.^3^, ^4^ Despite decreases in the rate of antipsychotic polypharmacy in Japan, rates remain the highest among Asian countries.^4^ Training and education programs for Japanese psychiatrists (e.g., the Effectiveness of Guidelines for Dissemination and Education Psychiatric Treatment Project^3^) may improve and minimize the rate of antipsychotic polypharmacy.

Our univariate analyses revealed significant differences in six metabolic parameters (i.e., BMI, sBP, dBP, TC, LDL‐C, and TG) as well as potential predictors (i.e., age, sex, type of care, CP‐equivalent dose, and antipsychotic type) among the four prescribed antipsychotic number groups. After controlling for the effects of these potential predictors, the associations between the number of antipsychotics prescribed and BMI and dBP remained significant using multiple regression analysis.

A recent study revealed that patients with schizophrenia are genetically protected against abnormalities in several metabolic parameters (i.e., BMI, sBP, HDL‐C, TG, and blood sugar), although the negative correlation between dBP and schizophrenia was not significant.^23^ These findings suggest that non‐genetic factors play an important role in the high prevalence of metabolic abnormalities in patients with schizophrenia. Our results indicate that the number of antipsychotics prescribed, rather than CP‐equivalent dose, is a risk factor for obesity and hypertension in patients with schizophrenia. Notably, a higher CP‐equivalent dose does not necessarily reflect antipsychotic polypharmacy. The higher the number of antipsychotics prescribed, the greater the likelihood that patients’ body weight and blood pressure will be affected because metabolic side‐effect profiles differ among antipsychotics.^21^ Indeed, the type of antipsychotics prescribed was associated with BMI and dBP, and aripiprazole was negatively correlated with BMI. Notably, meta‐analyses have demonstrated that aripiprazole augmentation of antipsychotics is associated with lower body weight and BMI.^24^, ^25^ Of the 2121 patients prescribed aripiprazole in the current study, 1208 (57.0%) received antipsychotic polypharmacy. The negative correlation between aripiprazole and BMI may reflect the protective effects of aripiprazole against antipsychotic‐induced weight gain. In contrast, olanzapine has been documented as the most BMI‐increasing antipsychotic.^21^ However, we found a negative correlation between olanzapine and BMI. This may be because, in real‐world practice, clinicians avoid prescribing olanzapine to patients with a high BMI. In addition, although olanzapine was correlated with dBP, risperidone was negatively correlated with dBP. A recent meta‐analysis reported that olanzapine and risperidone did not affect dBP.^26^ However, the number of studies on the effects of olanzapine and risperidone were seven and two, respectively, and the *I* ^2^ values for olanzapine and risperidone were 53.0% and 61.3%, respectively, indicating high heterogeneity across studies. Thus, to draw definitive conclusions about the effect of different antipsychotics on blood pressure, further prospective studies are needed.

Few studies have assessed the effects of the number of antipsychotics prescribed on metabolic parameters,^27^ although several studies have compared metabolic parameters between patients who received antipsychotic monotherapy and those who received polypharmacy.^9^, ^10^, ^11^, ^12^, ^13^ Our sample size (*n* = 19,675) was substantially larger than that of previous studies (*n* = 118–543),^9^, ^10^, ^11^, ^12^, ^13^ which enabled the detection of a small yet statistically significant adverse effect of the number of antipsychotics prescribed on BMI and dBP using both univariate and multivariate analyses. The differences in BMI and dBP among the groups were small, and these parameters were within normal limits for all four groups. Therefore, the clinical significance of our results should be interpreted with caution.

Our study had several limitations. First, we used a cross‐sectional design; therefore, we were unable to examine causal relationships. Second, antipsychotics were not randomly chosen. Thus, our results may have been influenced by selection bias. Third, we did not adjust for other potential confounding factors, such as duration of illness, concomitant use of other psychotropics (e.g., mood stabilizers), smoking status, and symptom severity.^21^, ^28^ Fourth, the data on physical illnesses and the use of medications to treat physical illnesses were not available. Approximately 7% of inpatients did not receive antipsychotics despite being hospitalized; some of these patients may not have been prescribed antipsychotics because of a physical illness. There were also differences in age among the four groups, of which those who were not treated with antipsychotics were the oldest. These patients were more likely to have physical illnesses and take more medications than the other groups. Therefore, we cannot exclude the possibility that our results were influenced by physical illnesses and the use of medications to treat physical illnesses.

In summary, our results suggest that the number of antipsychotics prescribed adversely affects BMI and dBP in patients with schizophrenia. However, large‐scale observational studies have reported that antipsychotic polypharmacy is more strongly associated with lower rehospitalization and mortality than antipsychotic monotherapy.^29^, ^30^ Therefore, the benefits and risks of antipsychotic polypharmacy remain controversial.^1^, ^2^ Nevertheless, we recommend clinicians avoid inappropriate antipsychotic polypharmacy, especially polypharmacy involving three or more antipsychotics.

## CONFLICTS OF INTEREST

Shin Ono received grant or research support from the Hospitals Bureau Niigata Prefecture Government and a Grant‐in‐Aid for Scientific Research I. Yutaro Suzuki received research support or honoraria from Janssen Pharmaceutical K.K., Mitsubishi Tanabe Pharma Co., Ltd., and Otsuka Pharmaceutical Co., Ltd. Norio Sugawara received grant or research support from the Grant‐in‐Aid for Young Scientists (B), Ministry of Education, Culture, Sports, Science and Technology, Japan Grant B, the Karoji Memorial Fund for Medical Research Grant, and the Senshin Medical Research Foundation. Norio Yasui‐Furukori received research support or honoraria from Astellas Pharma Inc., Dainippon Sumitomo Pharma Co., Ltd., Eli Lilly Japan, K.K., GlaxoSmithKline K.K., Janssen Pharmaceutical K.K., Meiji Seika Pharma Co., Mochida Pharmaceutical Co. Ltd., MSD K.K., Otsuka Pharmaceutical Co. Ltd., Pfizer Japan Inc., Takeda Pharmaceutical Co. Ltd., and Yoshitomi Pharmaceutical Industries. Kazutaka Shimoda received research support or honoraria from Daiichi Sankyo Co., Ltd., Dainippon Sumitomo Pharma Co., Ltd., Eisai Co., Ltd., Eli Lilly Japan, K.K., GlaxoSmithKline K.K., Meiji Seika Pharma Co., Ltd., Novartis Pharma K.K., Otsuka Pharmaceutical Co., Ltd., Pfizer Japan Inc., Shionogi & Co., Ltd., Takeda Pharmaceutical Co., Ltd., Tsumura & Co., Yoshitomi Pharmaceutical Industries, Ltd., Asahi Kasei Pharma Corporation, Astellas Pharma Inc., Janssen Pharmaceutical K.K., Kowa Pharmaceutical Co., Ltd., Meiji Seika Pharma Co., Mitsubishi Tanabe Pharma Corporation, Ltd., MSD K.K., Novartis Pharma K.K., and Ono Pharmaceutical Co., Ltd. Yuji Ozeki received research support or honoraria from Dainippon Sumitomo Pharma Co., Ltd., Eli Lilly Japan, K.K., Mochida Pharmaceutical Co. Ltd., Otsuka Pharmaceutical Co. Ltd., Eisai Co., Ltd., Shionogi & Co., Ltd., Daiichi Sankyo Propharma Co. Ltd., and Takeda Pharmaceutical Co. Ltd. Toshiyuki Someya received research support or honoraria from Asahi Kasei Pharma Corp., Astellas Pharma Inc., Daiichi Sankyo Co., Ltd., Dainippon Sumitomo Pharma Co., Ltd., Eisai Co., Ltd., Eli Lilly Japan, K.K., GlaxoSmithKline K.K., Janssen Pharmaceutical K.K., Meiji Seika Pharma Co., Ltd., Mitsubishi Tanabe Pharma Co., Ltd., Mochida Pharmaceutical Co., Ltd., MSD K.K., Otsuka Pharmaceutical Co., Ltd., Pfizer Japan Inc., Shionogi & Co., Ltd., Tsumura & Co., and Yoshitomi Pharmaceutical Industries. All other authors declare that they have no potential conflicts of interest.

## AUTHOR CONTRIBUTIONS

**Yuichiro Watanabe**: Conceptualization, Methodology, Writing — original draft, Writing — review & editing. **Shin Ono**: Conceptualization, Data curation, Formal analysis, Investigation, Validation, Methodology, Writing — original draft, Writing — review & editing. **Takuro Sugai**: Conceptualization, Data curation, Formal analysis, Investigation, Validation, Methodology, Writing — original draft, Writing — review & editing. **Yutaro Suzuki**: Conceptualization, Writing — review & editing. **Manabu Yamazaki**: Project administration, Resources, Writing — review & editing. **Norio Sugawara**: Conceptualization, Writing — review & editing. **Norio Yasui‐Furukori**: Conceptualization, Writing — review & editing. **Kazutaka Shimoda**: Conceptualization, Writing — review & editing. **Takao Mori**: Resources, Writing — review & editing. **Yuji Ozeki**: Conceptualization, Writing — review & editing. **Hiroshi Matsuda**: Resources, Writing — review & editing. **Kurefu Okamoto**: Resources, Writing — review & editing. **Toyoaki Sagae**: Conceptualization, Writing — review & editing. **Toshiyuki Someya**: Conceptualization, Funding acquisition, Project administration, Supervision, Writing — review & editing.

## ETHICS APPROVAL STATEMENT

This study was approved by the Institutional Review Board of the Japan Psychiatric Hospital Association.

## PATIENT CONSENT STATEMENT

All participants provided written informed consent.

## CLINICAL TRIAL REGISTRATION

Clinical trial registration information is not available.

## Data Availability

The data that support the findings of this study are available from the corresponding author upon reasonable request.
